# Application of sugar-free cola solution based on the ICOCFAS score in oral care of non-artificial airway patients with severe neurological conditions

**DOI:** 10.3389/fmed.2025.1684863

**Published:** 2025-11-20

**Authors:** Qingmei Wang, Yuanyuan Fan, Mei Chen, Ping Chen

**Affiliations:** 1 Department of Nursing, The Affiliated Huai’an No. 1 People’s Hospital of Nanjing Medical University, Huai’an, China; 2 Department of Neurology, The Affiliated Huai’an No. 1 People’s Hospital of Nanjing Medical University, Huai’an, Jiangsu, China

**Keywords:** ICOCFAS score, sugar-free cola, non-artificial airway, oral care, nursing

## Abstract

**Aim:**

This study aimed to explore the effect of using a coke solution based on the Intensive Care Oral Care Frequency Assessment Scale (ICOCFAS) score in oral care of patients with severe neurological conditions without an artificial airway.

**Methods:**

A total of 80 patients with severe non-artificial airways admitted to the neurological intensive care unit of our hospital from January 2023 to January 2024 were enrolled and divided into a control group (40 cases) and an observation group (40 cases). The control group was given routine oral care (0.9% saline gauze wipe). The observation group received oral care with gauze soaked in sugar-free cola solution and compared the family satisfaction of patients with complications of the modified Beck oral score between the two groups after implementing the program.

**Results:**

After the implementation of the program, the modified Beck oral score of patients in the observation group was lower than that of the control group on days 3, 5, and 7, with statistical significance (*p* < 0.05). The complication rate of the observation group was lower than that of the control group, with statistical significance (*p* < 0.05). The satisfaction degree of patients’ families in the observation group was higher than that of the control group, with statistical significance (*p* < 0.05).

**Conclusion:**

The application of sugar-free cola solution based on the ICOCFAS score in oral care of patients with severe neurological non-artificial airway has a good effect. It can significantly improve the oral health status of these patients, with a low complication rate, high safety, and high satisfaction of patients’ families, making it worthy of promotion and application.

## Introduction

Stroke patients in the neurological intensive care unit (NICU) suffer from loss of consciousness and neurological dysfunction, resulting in respiratory depression, coughing, swallowing reflex disorders, shortness of breath, and open-mouth breathing (1). Open-mouth breathing can lead to dryness of the patient’s mouth, throat, and lower respiratory tract. The self-cleaning ability of the mouth is reduced, resulting in complications such as cracked oral mucosa, bad breath, infection, and phlegm scab adhesion. In severe cases, the patient may asphyxiate due to airway secretions not being cleared in time and respiratory tract blockage, threatening the patient’s life (2). Oral care is an important part of clinical care for severe patients in the department of neurology. Oral care can keep the mouth clean and moist, and the most important thing is to remove dental plaque and microorganisms, and prevent complications such as oral infection (3).

At present, there is a lack of unified suggestions on the frequency of oral care in China, and it is not clear how to determine the specific frequency of oral intervention according to the evaluation results. Inadequate oral care cannot improve oral health. The frequency of oral care can be determined according to the scores of the Intensive Care Oral Care Frequency Assessment Scale (ICOCFAS) to prevent excessive frequency of oral care from increasing patients’ discomfort or even oral mucosal damage (4). Currently, there are many studies on oral gargling during hospitalization (5, 6), but there is no unified standard for the selection of oral care gargling for severe patients in the department of neurology. Although clinical trials specifically evaluating sugar-free cola in oral care are lacking, its pH ≈ 2.6 and phosphoric/carbonic acid content have been shown to dissolve tenacious proteinaceous masses in the gastrointestinal tract (7). The release of CO₂ bubbles further assists in mechanically dislodging debris and biofilm, thereby enhancing oral cleanliness. This dual mechanism-chemical dissolution and physical effervescence, may make it a potentially effective agent for oral care in patients with compromised oral self-cleaning ability. This study adopted targeted oral care with Diet Coke based on the ICOCFAS score.

## Methods

### Study design and patients

**Figure 1:**
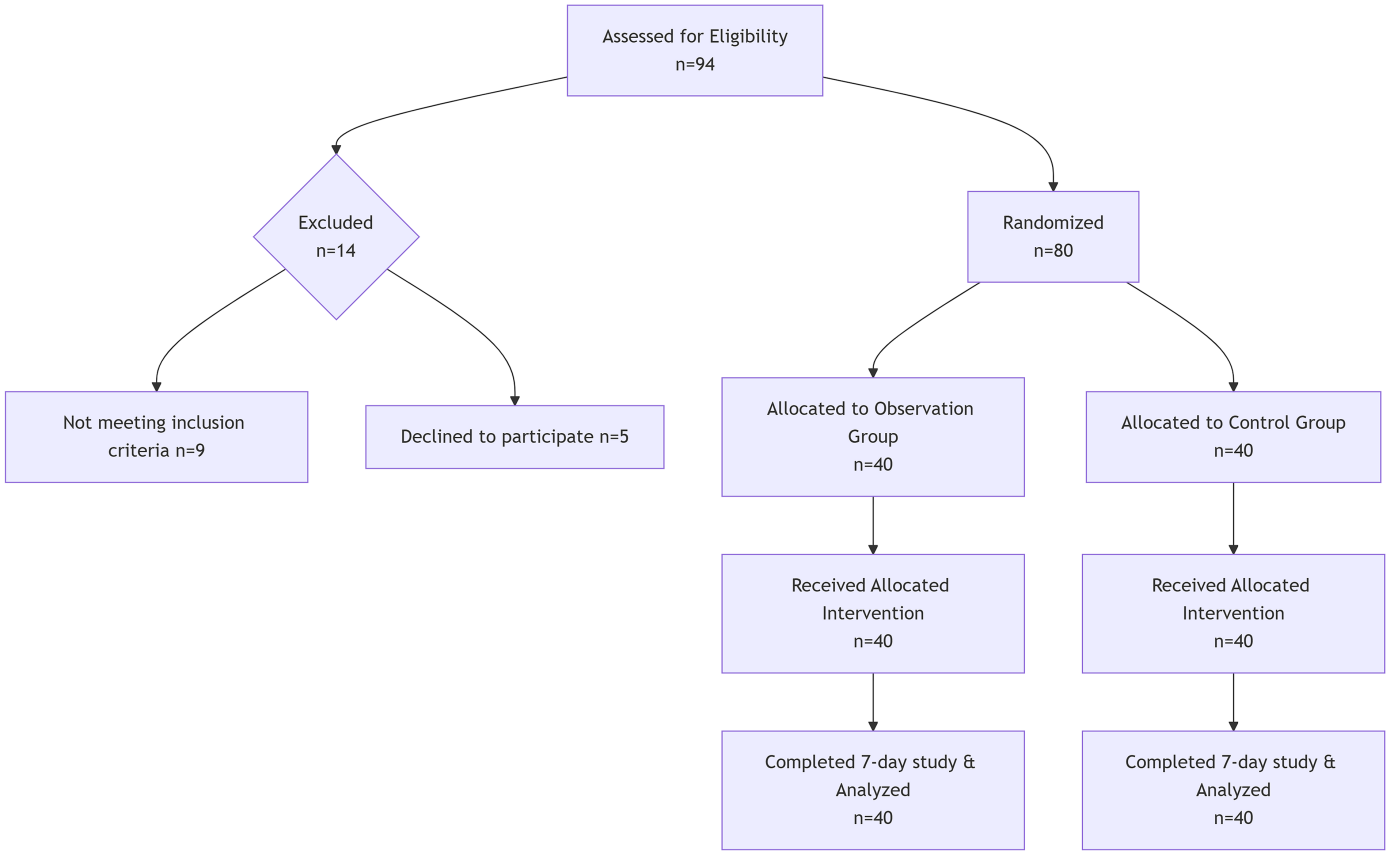
Flowchart of patient recruitment and follow-up.

This was a randomized controlled trial. A total of 80 patients with severe non-artificial airway admitted to the neurological intensive care unit of our hospital from January 2023 to January 2024 were enrolled. Patients were randomly assigned to either the control group (40 cases) or the observation group (40 cases) using a computer-generated random number sequence. The allocation was concealed using sealed opaque envelopes. The sample size was calculated using G*Power 3.1 based on previous research (8). With an effect size of 0.65, *α* = 0.05, and power = 0.80, the required sample size was 38 patients per group. We enrolled 40 patients per group to account for potential attrition (Figure 1).

There were 27 men and 13 women in the control group. Their ages ranged from 40 to 79 years, with an average age of 59.43 ± 10.43 years. There were 24 men and 16 women in the observation group. Their ages ranged from 42 to 81 years, with a mean of 60.23 ± 10.76 years. There was no statistical significance in the general data for the two groups (*p* > 0.05).

### Inclusion and exclusion criteria

The inclusion criteria included: (1) combined with clinical manifestations and head CT and/or MRI diagnosis, the diagnosis meets the relevant criteria in the Chinese Classification of Cerebrovascular Diseases 2015, (2) patients with severe non-artificial airway in the neurology department, and (3) patients or family members voluntarily participated and signed a written consent.

The exclusion criteria included: (1) the duration of hospitalization is less than 7 days, (2) patients with an artificial airway, (3) there were oral diseases such as cracked lips, oral ulcers, and oral infections when hospitalized, and (4) Patients and family members do not agree with this method of oral care.

### Interventions

The control group was given 0.9% saline gauze (20 cm × 30 cm) to wipe the oral cavity with the conventional oral care method by nurses, and the operation method was referred to the seventh edition of Basic Nursing (9), once in the morning and once in the evening.

In the observation group, the Chinese version of the Intensive Care Oral Care Frequency Assessment Scale (ICOCFAS) (10) was used to implement targeted oral care for patients every day according to the oral score. Oral care should be performed every 12 h with an ICOCFAS score ≤ 9, every 8–12 h with an ICOCFAS score of 10–19, every 6 h with an ICOCFAS score of 20–29, and every 4 h with an ICOCFAS score of ≥30. The sugar-free cola solution used was undiluted, carbonated Coca-Cola Zero, and stored at room temperature. Sterile gauze (20 cm × 30 cm) was soaked in the solution until fully saturated but not dripping, and then used to wipe all surfaces of the oral cavity-including teeth, cheeks, tongue, and hard palate-following the brushing procedure recommended by the American Dental Association (11). After wiping with cola-soaked gauze, the mouth was re-wiped with 0.9% saline-soaked gauze to remove residual acidity and secretions. During the wiping, nurses paid attention to the mouth, and the negative pressure suction device was used to wash the oral secretions of the patient in time to avoid aspiration.

The airway care team members of the department are responsible for supervising the oral health assessment and oral care operation procedures every day, and the department gives feedback and rectification every month.

### Observation indicators

Oral health status was assessed using the modified Beck oral rating scale and was evaluated by a specially-assigned person before the first oral care on days 3, 5, and 7 after the implementation of the reform program. The assessments were performed by two senior nurses from the airway care team who were not involved in providing oral care to the study participants. Both assessors received standardized training on the use of the modified Beck scale to ensure consistent evaluation. Although blinding of the nursing staff administering the care was not possible due to the nature of the interventions, the outcome assessors were blinded to the patient’s group allocation throughout the study period to minimize assessment bias. The Beck oral assessment tool was developed and designed by Beck (12) for the oral care of cancer patients, and was revised by Ames et al. into an improved Beck oral score for ICU patients (13), which was finally improved into five evaluation indicators: mouth, tongue, teeth, oral mucosa and gums, and saliva, with a total score of 5–20; the higher the score, the worse the oral function of the patient.

The occurrence of complications, including dry lips, bad breath, fungal infection, and oral ulcer in the two groups was observed and recorded.

Family satisfaction with patients in the two groups was recorded. The self-made patient satisfaction evaluation table was used for evaluation, including six items: (1) Whether your bed nurse often listened to your chief complaint and could meet your needs in time. (2) Does your bed nurse pay timely attention to your oral cleanliness? (3) Whether your bed nurse gives you oral care on time. (4) Does your bed nurse pay attention to your oral comfort? (5) Does your bed nurse pay attention to your oral complications? (6) Are you satisfied with the effect of oral care during your stay at the hospital? Likert five scores were adopted, which were “very satisfied,” “satisfied,” “general,” “dissatisfied,” and “very dissatisfied” (14). Satisfaction rate = (very satisfied + satisfied)/(very satisfied + satisfied + general + dissatisfied + very dissatisfied) × 100%. A satisfaction score above 85% is considered satisfactory.

### Statistical methods

SPSS 21.0 software was used to analyze the data, and a *p*-value of <0.05 indicated statistically significant differences. The statistical data were represented by [n (%)], and the χ^2^-test was used for comparison between groups. After the K–S test, the measurement data were in line with a normal distribution, represented by mean ± SD, and an independent sample t-test was performed to compare between groups.

## Results

### Comparison of the oral health status between the two groups

The modified Beck oral scores of the two groups were compared before the implementation of the program on days 3, 5, and 7 of oral care, and the modified Beck oral scores of the observation group were lower than those of the control group. The difference was statistically significant (*p* < 0.05) (Table 1).

**Table 1:** Modified Beck oral scores on days 3, 5, and 7 after the protocol modification (mean ± SD, points).

Group	Example number	Day 3	Day 5	Day 7
Observation group	40	9.50 ± 2.08	9.40 ± 2.75	9.05 ± 2.90
Control group	40	15.10 ± 2.54	14.85 ± 2.47	13.05 ± 3.03
*t*	–	10.798	9.326	6.033
*P*	–	<0.001	<0.001	<0.001

Compared with the pre-intervention procedure. ^*^p < 0.05.

### Comparison of complications between the two groups

The incidence of complications in the observation group was lower than that in the control group after interventions. The difference was statistically significant (*p* < 0.05) (Table 2).

**Table 2:** Complications in the two groups [n (%)].

Group	Example number	Xerocheilia	Oral odor	Mycotic infection	Dental ulcer	Incidence (%)
Observation group	40	2	1	0	1	4(10%)
Control group	40	3	6	3	2	14(35%)
χ^2^ value	–					7.169
*P*	–					0.007

Throughout the study period, the oral mucosa of all patients was closely monitored. No adverse events related to mucosal irritation, erosion, or damage attributed to the application of the sugar-free cola solution were observed in the observation group. The complications recorded (Table 2) were those commonly associated with poor oral hygiene in critically ill patients.

### Comparison of family satisfaction between the two groups

After the implementation of the program, family satisfaction was significantly higher in the observation group than in the control group, and the difference was statistically significant (*p* < 0.05), as shown in Table 3.

**Table 3:** Comparison of family satisfaction between the two groups [n (%)].

Group	Example number	Very satisfied	Satisfied	General satisfaction	Discontent	Very dissatisfied	Degree of satisfaction (%)
Observation group	40	25	10	3	2	0	35(87.5%)
Control group	40	17	10	1	2	2	27(67.5%)
χ^2^ value	–						25.208
*P*	–						<0.001

## Discussion

The majority of the patients with severe stroke have dysphagia because respiratory function is inhibited due to consciousness disturbance, and open-mouth breathing occurs. Coupled with the application of dehydrating agents, saliva secretion is reduced, which can easily lead to complications such as cracked lips, oral mucosa damage, bleeding, infection, and phlegm crusting and adhesion. Oral mucosa can effectively block the invasion of pathogens, but patients with severe neurological conditions have reduced resistance and impaired oral mucosal function, which cannot effectively prevent the colonization of pathogens (1). Therefore, effective, standardized, and scientific oral care plays a very important role in the prevention and recovery of complications in patients with severe neurological non-artificial airways.

At present, a variety of mouthwash products and oral care products are used clinically. Mouthwash is an important factor affecting the effectiveness of oral care (15, 16). In traditional oral care, normal saline is mainly used for oral cleaning. Although it can remove some pollutants in the mouth and achieve certain cleaning and wetting effects, its ability to remove adherent oral phlegm scabs is limited, and some impurities remain, resulting in longer nursing time. It is also not effective in the prevention of complications such as bad breath, oral ulcer, and oral infection (17). Domestic Ou et al. confirmed that cola can dissolve the attachment attached to the wall of the nasointestinal tube and play a role in eliminating clots (18). In this study, the pH of 2.6 of Diet Coke can acidify oral secretions, such as phlegm crusting and release CO_2_, thus playing a dissolving role. After wiping the mouth with Diet Coke, use 0.9% normal saline gauze to wipe the mouth in the same way, thus effectively removing oral secretions, phlegm crusting, and bacteria, and keeping the mouth clean and moist. Ultimately, this leads to improved oral health, the elimination of unpleasant oral odors, and the prevention of oral complications.

A study has shown that the higher the score of the Intensive Care Oral Care Frequency Assessment Scale (ICOCFAS), the higher the frequency of oral care required by patients (4). This study is based on the ICOCFAS score to guide clinical practice and determine the frequency of oral care for non-artificial airway patients with severe neurological conditions. The higher the score, the higher the frequency of oral care, and the lower the score, the lower the frequency of oral care. The results showed that when oral care was given to patients with severe neurological conditions according to the ICOCFAS score, nurses could take the initiative to pay attention to patients’ oral health status, timely detect oral problems, effectively improve patients’ oral health status, and reduce the number of oral bacteria, oral odor, oral mucosal damage, and other complications. At the same time, it obviously reduces the waste of nursing manpower, material resources, and financial resources.

The improved Beck oral score can systematically and dynamically evaluate the oral health status of patients. The lower the score, the better the oral health status. Comprehensive cleaning of the patient’s mouth and the implementation of personalized oral care frequency can ensure the oral health level of the patient, inhibit the proliferation of oral bacteria, and thus improve the oral health status of the patient (19). In this study, targeted oral care was implemented with sugar-free cola gauze based on the ICOCFAS score. The results showed that the improved Beck oral score of the observation group was significantly lower than that of the control group before the first oral care on days 3, 5, and 7 after the implementation of the program (*p* < 0.05). The incidence of complications in the observation group was significantly lower than in the control group (*p* < 0.05). The oral care based on the ICOCFAS score is highly targeted, and wiping the mouth with sugar-free cola gauze can effectively remove the secretions and bacteria in the mouth, maintain the microenvironment in the mouth, prevent oral diseases, and further improve the treatment effect and promote the recovery of patients.

Patients with severe neurological disorders have poor oral self-cleaning ability due to consciousness disorders, and often have complications such as bad breath, cracked lips, oral ulcers, and lung infections, which their families worry will affect the rehabilitation of patients. The results of this study showed that the satisfaction of family members in the observation group was higher than that in the control group (*p* < 0.05), suggesting that targeted oral care with diet cola in non-artificial airway patients with severe neurological symptoms according to ICOCFAS score could not only effectively remove oral secretions, keep the oral cavity clean and moist, but also reduce the occurrence of complications. It was unanimously affirmed by the patients and their families.

This study has several limitations. First, it was conducted in a single center with a relatively small sample size, which may affect the generalizability of the findings. Future multi-center studies with larger samples are needed. Second, while robust randomization and allocation concealment were used, the nature of the intervention made blinding of the care providers impossible, which is a common limitation in pragmatic trials of this kind. Finally, the long-term safety of repeatedly using an acidic solution such as diet cola, even with the neutralizing step of saline wiping, was not fully explored in this short-term study. Investigations with longer follow-up and more sensitive assessments of oral mucosa and enamel are recommended.

## Conclusion

Targeted oral care with diet cola in non-artificial airway patients with severe neurological conditions according to the ICOCFAS score can significantly improve oral cleanliness and effectively reduce complications such as cracked lips, bad breath, fungal infection, and oral ulcers. The method is simple, convenient, safe, and effective, saves manpower, material, and financial resources, and has strong applicability, which is suitable for promotion and application to severe clinical patients. In the next step, we will expand the clinical sample size and apply it to relevant alliance units to comprehensively analyze the clinical benefits of the modified oral care method, and develop evidence-based personalized oral care for critically ill patients, which has important clinical significance for nosocomial infection prevention and control and public health burden.

## Data Availability

The original contributions presented in the study are included in the article/supplementary material, further inquiries can be directed to the corresponding author.
